# BET Proteins as Attractive Targets for Cancer Therapeutics

**DOI:** 10.3390/ijms222011102

**Published:** 2021-10-14

**Authors:** Joanna Sarnik, Tomasz Popławski, Paulina Tokarz

**Affiliations:** 1 Department of Rheumatology, Medical University of Lodz, 90-050 Lodz, Poland; joanna.sarnik@umed.lodz.pl; 2 Department of Molecular Genetics, Faculty of Biology and Environmental Protection, University of Lodz, 90-236 Lodz, Poland; tomasz.poplawski@biol.uni.lodz.pl

**Keywords:** BET, BETi, cancer, DNA repair, homologous recombination, transcription

## Abstract

Transcriptional dysregulation is a hallmark of cancer and can be an essential driver of cancer initiation and progression. Loss of transcriptional control can cause cancer cells to become dependent on certain regulators of gene expression. Bromodomain and extraterminal domain (BET) proteins are epigenetic readers that regulate the expression of multiple genes involved in carcinogenesis. BET inhibitors (BETis) disrupt BET protein binding to acetylated lysine residues of chromatin and suppress the transcription of various genes, including oncogenic transcription factors. Phase I and II clinical trials demonstrated BETis’ potential as anticancer drugs against solid tumours and haematological malignancies; however, their clinical success was limited as monotherapies. Emerging treatment-associated toxicities, drug resistance and a lack of predictive biomarkers limited BETis’ clinical progress. The preclinical evaluation demonstrated that BETis synergised with different classes of compounds, including DNA repair inhibitors, thus supporting further clinical development of BETis. The combination of BET and PARP inhibitors triggered synthetic lethality in cells with proficient homologous recombination. Mechanistic studies revealed that BETis targeted multiple essential homologous recombination pathway proteins, including RAD51, BRCA1 and CtIP. The exact mechanism of BETis’ anticancer action remains poorly understood; nevertheless, these agents provide a novel approach to epigenome and transcriptome anticancer therapy.

## 1. Introduction

Cell identity and its proper functioning are determined by the transcriptome. The single-cell transcriptome is regulated by tens of thousands of promoter and enhancer regions and a few hundred super-enhancer—clusters of enhancers binding master transcription factors and mediators [1,2,3]. The control of the transcriptome is even more complex given epigenetic changes, including noncoding RNA synthesis, DNA methylation and histone modification. Histone acetylation at lysine residues is a reversible and highly dynamic modification frequently disturbed in cancer cells, making it an attractive anticancer therapy target. Histone acetylation is under the control of histone acetyltransferases (HATs), which are the “writers”, bromodomain (BRD) proteins, which are the “readers”, and histone deacetylases (HDACs) and sirtuins, which together are the “erasers”.

Transcription dysregulation is a hallmark of cancer. Loss of transcriptional control leads to changes in gene expression, which could be a driving force behind carcinogenesis. Defective DNA damage response (DDR) and repair pathways are cancer cells’ common features that trigger disease initiation and progression. The efficacy of DNA damage repair is provided by the proper structure of repair proteins and a sufficient amount of DDR and repair pathway members. Alterations in DDR and repair genes’ transcription may have significant consequences for carcinogenesis, response to treatment and acquisition of resistance. In this review, we discuss the role of epigenetic readers in the transcriptional control of DNA repair genes and the implications for carcinogenesis and anticancer therapy.

## 2. BET Proteins’ Function

**Figure 1:**
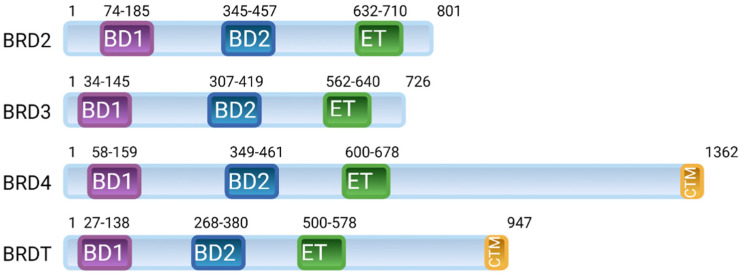
Arrangement of domains in the human bromodomain and extraterminal domain (BET) family of proteins; BRD2, BRD3, BRD4 and BRDT. BET proteins contain two N-terminal bromodomains (BD1 and BD2), an extraterminal domain (ET) and a C-terminal motif (CTM).

The bromodomain and extraterminal domain (BET) family belongs to BRD proteins and comprises four evolutionarily conserved members, including ubiquitously expressed BRD2, BRD3 and BRD4 and a testis-specific BRDT [3,4]. The BET family is characterised by the presence of two N-terminal bromodomains, BD1 and BD2, and an extraterminal domain (ET) (Figure 1) [4,5,6]. BRD4 and BRDT also contain a C-terminal motif (CTM) that facilitates the recruitment of transcriptional regulators, including the positive transcription elongation factor (P-TEFb). Among these domains, BD1 and BD2 have a conserved sequence of 110 amino acids that creates a hydrophobic binding pocket for acetylated lysine residues on histones and other proteins [7,8]. BD domains are composed of a four-helix bundle (αZ, αA, αB and αC) and two loops, ZA and BC. The ET domain is a conserved region of ~80 amino acids that recruits transcription effector proteins [9,10,11,12]. The CTD domain is a conserved region of ~40 amino acids responsible for the recruitment of P-TEFb [13,14].

BRD2 (alias RING3 or FSRG1) is a serine/threonine kinase that is a component of a mediator—a multiprotein complex functioning as a transcriptional co-activator of RNA polymerase II (Pol II) [15]. BRD2 recruits the E2F-1 and E2F-2 transcription factors and assists in the Pol II-mediated transcription in hyperacetylated chromatin, thereby coupling histone acetylation to transcription [16,17,18]. Along with E2F proteins, BRD2 activates the promoters of several cell cycle regulatory genes, including cyclin A, cyclin D11 and cyclin E [18,19]. Moreover, BRD2 was shown to exert histone chaperone activity [17].

BRD3 (alias ORFX) is a serine/threonine kinase that facilitates Pol II transcription through hyperacetylated nucleosomes independent of P-TEFb [17]. BRD3 might cooperate with BRD4 to recruit P-TEFb to the chromatin and thus promote transcriptional activation [20]. BRD3 binds the GATA1 transcription factor in an acetylation-dependent manner and facilitates stable association with chromatin [21,22]. BRD3 together with BRD4 are characterised to be required for IL-1β- or TNF-α-induced transcription [20].

BRDT (bromodomain, testis-specific, alias BRD6) is specifically expressed in testis and ovaries [23]. BRDT modulates gene expression as part of the splicing and participates in chromatin remodelling [7,24]. BRDT interacts with acetylated histone H4 and might assist in the removal of acetylated histones during spermatogenesis [8].

**Figure 2:**
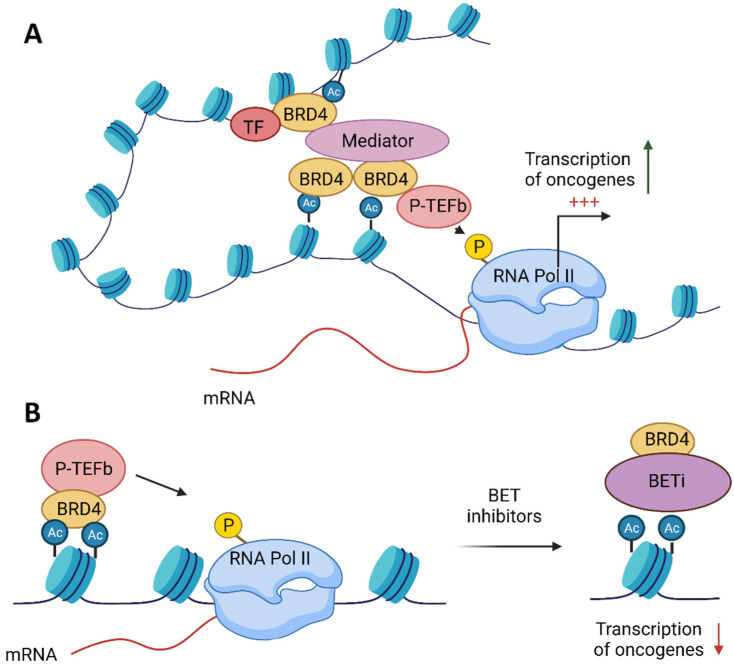
Function of the bromodomain and extraterminal domain 4 (BRD4) protein and the role of its inhibition in anticancer therapy. (**A**) BRD4 binds to acetylated histones and, via its C-terminal motif (CTD), facilitates the recruitment of the positive transcription elongation factor (P-TEFb) to chromatin. BRD4 enables the interaction among transcription factors (TF), the mediator complex and P-TEFb, which result in the phosphorylation of RNA polymerase II (RNA pol II) on serine 2 of its C-terminal domain, thereby stimulating transcriptional elongation. (**B**) BET inhibitors (BETis) dissociate BRD4 from acetylated histones, thus repressing the transcription of oncogenes.

BRD4 (alias MCAP or Hunk1) is a serine kinase and is the most extensively studied member of the BET family. It is a chromatin-binding factor with a preference for acetylated Lys-14 on histone H3 and Lys-5/12 on H4 [6]. BRD4 comprises three splice isoforms, A, B and C, and only isoform A contains the CTD domain [25]. BRD4 isoform A has a well-established role in transcriptional modulation, mainly as a transcriptional co-activator of P-TEFb, which stimulates Pol II transcription (Figure 2) [13,14]. BRD4 binding facilitates P-TEFb recruitment to chromatin. Moreover, a core component of P-TEFb was identified as cyclin-dependent kinase-9 (CDK9)—a target in chronic lymphocytic leukaemia and necessary for MYC-mediated transcription regulation [26,27,28]. BRD4 marks transcriptional start sites of growth-associated genes at the M/G1 transition throughout mitosis [29].

## 3. BET Proteins in Cancer

BET and, in particular, BRD4 have been implicated in human diseases, especially cancer. Clinical research provides direct evidence of the oncogenic roles of BRD3 and BRD4 [30,31,32]. *BRD3* and *BRD4* genes translocation t(15;19) leads to a fusion protein with nuclear protein in testis (NUT), which causes a rare, but aggressive form of human squamous carcinoma. The BRD-NUT oncoprotein contributes to the carcinogenesis of NUT midline carcinoma (NMC). Genome-wide sequencing revealed that *BRD3/4*-*NUT* rearrangements are major oncogenic drivers of NMC [33]. The BRD4-NUT oncoprotein blocks differentiation in NMC cells by maintaining MYC expression, and BRD4-NUT suppression results in NMC cell differentiation [32,34]. In addition to BRD-NUT-driven malignancies, the *BRD4* gene was found to be amplified across 20 types of common cancers [35]. Furthermore, BRD4 levels are upregulated in various tumours, leading to aberrant expression of growth-promoting genes and transcription factors [36,37,38]. A primary downstream target of BRD4 is MYC—a member of the myc family of transcription factors encoded by the proto-oncogene, which is frequently deregulated in cancer [37,39]. The first indication that MYC regulation might depend on BET came from the observation that P-TEFb is not recruited to the *MYC* locus in BRD4 knockdown cells, suggesting that BRD4 is critical for *MYC* transcription [13]. Besides MYC, BET influences the expression of other transcription factors such as ERG, c-Myb, E2F1 and nuclear factor κB (NF-κB) (reviewed in [40]).

## 4. BET Inhibitors Target MYC

MYC has long been considered a compelling therapeutic target because of its role in a range of human malignancies. Despite the urgent need to suppress this oncogenic driver, MYC had been deemed “undruggable” due to a large protein–protein interaction interface and its lack of a deep protein pocket [41]. The advent of BET inhibitors (BETis) was motivated by previous research establishing BRD4 as a promising anticancer target [13]. The first two inhibitors that competitively bind the acetyl-lysine recognition motif (bromodomain) of BET proteins were presented in 2010. I-BET (GSK525762), a benzodiazepine derivative, targets the expression of inflammatory genes [42]. JQ1, a novel thieno-triazolo-1,4-diazepine, selectively inhibits all BET family members: BRD2, BRD3, BRD4 and BRDT [39]. JQ1 displaces BRD4 from nuclear chromatin in cells and induces differentiation in NMC cells. A study in NMC xenograft tumours confirmed the antiproliferative and pro-apoptotic effects of JQ1. The publications on the first BETis and the accessibility of JQ1 accelerated the preclinical studies. Soon, it was demonstrated that BETis provide effective treatment against multiple myeloma (MM), acute myeloid leukaemia (AML), Burkitt’s lymphoma and mixed-lineage leukaemia (MLL)-rearranged leukaemias [36,37,38,43]. All the above papers emphasised the mode of the action of BETis, at least in part, due to the suppression of MYC transcription and cell cycle arrest. Most attention has been paid to selective targeting of critical oncogenic drivers such as *MYC* in multiple tumour types by BETi. The mechanism of *MYC* suppression by BETi is mainly conducted by the loss of BET at the *MYC* super-enhancer, a regulatory DNA fragment comprising multiple enhancers binding diverse transcription factors to provide the gene expression necessary for cell identity [1,3]. In many instances, BETis suppressed *MYC* transcription in a dose-dependent manner [36,37]. This downregulation is particularly pronounced in *MYC*-amplified tumours [44]. Although in most cases, there is a positive correlation between *MYC*-amplification and sensitivity to BETis, surprisingly, tumours lacking *MYC* amplification respond to the BETi treatment similarly to those with *MYC* amplification, suggesting that there are other targets within a cell for BETis [44,45,46]. Clinical trials confirmed that MYC expression fails to predict sensitivity to BETis in haematological malignancies and thus questioned its validity as a predictive biomarker [47]. The complexity between *MYC* expression and response to BETis may be due to the multiple mechanisms of the BET proteins’ function.

## 5. BET Inhibitors in Clinical Trials

Although JQ1 was a promising drug candidate in preclinical studies, with excellent oral bioavailability and good pharmacokinetics, its short half-life (one hour in vivo) limits its clinical application [39]. Nevertheless, JQ1 was the first compound, together with I-BET, that allowed for the mechanistic study of BET proteins’ functions and their oncogenic potential. Over the last few years since the development of JQ1 in 2010, a few new BETis have been synthesised and evaluated in clinical studies (Table 1; reviewed in [48,49]). 

As described above, the oncogenic role of BRD was initially described in NMC, which is driven by *NUT* translocation, usually involving *BRD3* or *BRD4*. The BRD-NUT fusion oncoprotein results in aberrant BRD activity. The promising data from preclinical studies on the efficacy of BETis against NMC led to a first published clinical trial of BETi, OTX015 (MK-8628) [50]. This first proof-of-concept small-scale study evaluated the antitumor activity of OTX015, a JQ1 analogue, in four advanced-stage NMC patients with confirmed BRD4-NUT fusions. Three out of four patients responded to the treatment, including two patients with rapid tumour regression and a third with disease stabilisation (SD). Two patients achieved an overall survival (of 18 mo and 19 mo) longer than the median survival of 6.7 mo [50,51]. Complementary studies on NMC showed that eight out of ten patients responded to OTX015 at 80 mg once daily, including three patients with partial response (PR) and three patients with SD for 1.8–8.4 mo [52]. Confirmed PR (two out of nineteen patients) and SD (seven out of nineteen patients) has been observed for NMC patients treated with GSK525762 [53]. Although clinical responses in NMC patients to BETis have been observed, these trials showed less-than-anticipated efficacy coming from preclinical observations. 

Most BETis achieve the cytostatic effect as a single agent at an approximate concentration of 500 nM across different cancer cell lines (reviewed in [54]). Such an effective concentration in preclinical studies translates into high concentrations in clinical settings, approximately 80 mg daily. Most clinical trials reported severe dose-limiting toxicities (DLTs), including thrombocytopenia, neutropenia, anaemia, gastrointestinal disorders, hyperbilirubinaemia, fatigue, headache and pain [47,52,55]. Hopefully, the adverse events were manageable and reversible. The first two clinical trials on OTX015 were published in the same issue of *The Lancet Haematology* journal in 2016. Both studies were aimed to establish the recommended dose of OTX015 in patients with acute leukaemia [55], lymphoma and MM [47]. Both studies led to the same recommendation of a drug dose of 80 mg once daily for single-agent oral OTX015 use in patients on a 14 d on and 7 d off schedule. Thrombocytopenia was the most reported DLT (96% patients). Due to the reversibility of thrombocytopenia after treatment interruption, the DLT was attributed to grade 4 [47]. No DLTs were recorded until 160 mg/d in five patients with acute leukaemia [55]. Complementary phase II studies recommended a dose of OTX015 in patients with castrate-resistant prostate cancer (CRPR), NMC and nonsmall-cell lung cancer at 80 mg once daily with continuous dosing [52]. DLTs included ALT, hyperbilirubinemia and thrombocytopenia grade 3 and 4. 

AZD5153 is a bivalent BETi that was optimised to interact with both bromodomains of BRD4 [56]. Preclinical studies showed that AZD5153 is effective as an anticancer agent at significantly lower concentrations than most BETis [57,58]. AZD5153 inhibited the growth of haematological, prostate and thyroid cancer cell lines at a concentration <150 nM [57,58,59]. Most haematological cell lines responded to the treatment at a concentration <25 nM [57]. Importantly, AZD5153 demonstrated a cytotoxic effect at a concentration 100 nM and tumour regression at concentrations 2.5–10 mg/kg for AML and prostate cancer xenografted tumours [57,58,59]. AZD5153 progressed to clinical trial, which reported similar DLTs as for OTX015, indicating that although BETis’ chemical structure is diverse, toxicities may be shared across BETis [60]. A dose escalation study of a new BETi, BAY1238097, in eight patients with solid cancers was prematurely terminated due to DLTs [61]. ABBV-075 is a BETi that strongly inhibits BRD2, BRD4 and BRDT, but not BRD3 (K_i_ = 1–2.2 nM). A large screen of 147 haematological and solid cancer cell lines demonstrated that ABBV-075 inhibited cell proliferation at concentrations similar to AZD5153 and was more effective against haematological than solid tumours malignancies [62]. A dose escalation study recommended ABBV-075 monotherapy at a concentration of 1.5 mg for the daily schedule, 2.5 mg for 4/7 and 3 mg for 3/7 for patients with advanced solid tumours [53]. Among 71 patients with solid tumours, 26 (43%) had SD. Despite a lower effective concentration (approximately 10-fold lower than OTX015) and a higher selectivity toward BET, ABBV-075 led to DLTs. Consistent with previous clinical trials on BETis, thrombocytopenia, gastrointestinal effects and hypertension were among the most common adverse events, which all were reversible. Since no selective BETis have entered clinical trials, it is difficult to foresee their outcome. It is expected that selective BETis would maintain their efficacy, but minimise the side effects [63]. To explore the individual functional contributions of BD1 and BD2 in biology and therapy, selective BD1 and BD2 inhibitors have been developed: GSK778 and GSK046 (termed iBET-BD1 and iBET-BD2, respectively) [63]. iBET-BD1 phenocopies the effects of pan-BET inhibitors in cancer models, whereas iBET-BD2 is predominantly effective in inflammatory and autoimmune disease models.

## 6. Resistance to BET Inhibitors

The effectiveness of anticancer therapies can be limited by primary and acquired resistance [73]. Preclinical and clinical studies presented varying sensitivities of cancer cells to BETis, suggesting that drug resistance could, at least partially, contribute to this effect. Until now, the resistance to BETis has not been attributed to *BRD2/3/4* mutations. Two studies indicated that the WNT signalling pathway is implicated in AML resistance to BETis [74,75]. Possible mechanisms of resistance to BETis encompass AMPK-ULK1-mediated autophagy in AML [76,77], NF-κB in colorectal cancer [78], PP2A phosphatase and BCL2L1/BCL-X in breast cancer [79], the GLI2-dependent Hedgehog pathway in pancreatic cancer [80] and kinome reprogramming in ovarian cancer [81], among others. Further studies demonstrated multiple mechanisms of resistance to BETis in solid tumours, including triple-negative breast cancer (TNBC), CRPC, and lung cancer [82,83,84]. The multitude of resistance mechanisms in diverse cancer models indicates that sensitivity to BETis might be cancer-cell-type-dependent. Further clinical studies, possibly in combination, will need to address this issue.

## 7. BET Inhibitors in Combination Therapy

Clinical studies demonstrated that BETis generally cause modest anticancer activity, predominantly due to the cytostatic effect, and evoke DLTs and adaptive resistance. BETi monotherapy can induce complete remission in NMC and non-NMC cancer types, particularly in haematological cancers [47,51,55,85]. Considering that (1) these remissions are often short-lived, (2) primary and acquired resistance emerge and (3) DLTs arise, the combination of BETis with other conventional and targeted therapies can provide meaningful clinical benefits. Emerging data from preclinical studies revealed that BETis have improved activity when used in combination therapy. In addition, BETis have been synergised with different classes of compounds in various tumour types, including solid tumours and hematologic malignancies (Table 2) [49]. Combinatorial treatment with molecularly targeted agents such as inhibitors of phosphoinositide 3-kinase (PI3K), extracellular signal-regulated kinases (ERK) and poly(ADP-ribose)polymerase (PARP) was beneficial. Strong synergy has been observed with histone deacetylase HDAC inhibitors (HDACis) in solid tumours and hematologic malignancies, indicating that this combination could be generalisable. A benefit has been observed when combining BETis with kinase inhibitors, including ALK, BTK, CDK, PLK1, JAK2 and PIKK. Since BETis generally induce a cytostatic effect, their complementation with apoptotic triggers, such as BCL-2 inhibitors, proved successful, and several clinical trials evaluated their efficacy. Additionally, BETis demonstrated synergistic activity in combination with immune system modulators, chemotherapeutics, hormone therapeutics and other epigenetic drugs. Some of the combinations mentioned above are discussed below.

Large-scale combinatorial screening with BETis and ~1900 compounds from the Mechanism Interrogation PlatE (MIPE) library in two *MYC*-amplified neuroblastoma cell lines identified PI3K inhibitors among the most synergistic combinations [86]. This combination was validated in a diverse panel of neuroblastoma cell lines and in vivo, including a patient-derived xenograft mouse model of *MYCN*-amplified neuroblastoma. Moreover, the combination of BETis and PI3K inhibitors proved effective in various cancer types (Table 2). Several studies reported that inhibition of the PI3K pathway could overcome the primary and acquired resistance to BETis [81,86]. Among the downstream effectors of the PI3K pathway is mTOR, which regulates cell growth, proliferation and survival [87]. BETis and mTOR inhibitors combined resulted in a synergistic antitumour effect in vitro and in vivo (Table 2). This combination proved successful in a diverse panel of cancer cells, including breast cancer, glioblastoma, lymphoma and osteosarcoma, suggesting that this is worth being further evaluated as cancer-type-independent treatment. The MEK-ERK signalling pathway intertwines with the PI3K-mTOR pathway, and alterations in both signalling pathways are frequently observed in cancer cells [88]. Gene expression profiling revealed that the MEK-ERK pathway was upregulated after BETi treatment, suggesting its participation in acquired resistance [89]. Indeed, the combination of BETis and MEK-ERK inhibitors predominantly exhibits synergy in most cancer cell lines of various origins (Table 2). 

It is well accepted that epigenetic status is already widely altered at the cancer initiation stage [90]. Given the importance of epigenetic changes in carcinogenesis, epigenome-targeted therapy has been considered a promising strategy for anticancer treatment. The efficiency of anticancer treatment is generally higher when epigenome-oriented drugs are applied in combination than as a monotherapy. Histone acetylation is a dynamic and reversible process that makes it a high-priority therapeutic target. HDACs regulate the level of acetylation of lysine residues on histone tails, and HDACis are potent agents that disrupt this modification and are used clinically in anticancer treatment. Several studies have reported the increased efficacy of HDAC and BET dual inhibition in cancer cells (Table 2). HDACis and BETis have similar antitumorigenic effects, e.g., HDACis suppresses *MYC* expression and its target genes [91]. The synergistic effect of combined HDACi and BETi treatment is challenging to explain since HDACis would be expected to increase global histone acetylation levels, thus providing a platform for BET binding, whereas BETis would prevent BET–chromatin interaction [92]. HDACs were shown to have variable activity depending on the genomic region. HDACis have limited activity at promoters, whereas a robust change in acetylation was observed at gene bodies. It was suggested that HDACis might redistribute BET from promotors and enhancers toward gene bodies. The suppression of chromatin interaction with BET would explain the similar cellular effects evoked by HDACis and BETis and the synergistic effect of their combination. A mechanistic study revealed that the cytotoxic effect induced by the cotreatment with JQ1 and HDACi was attributed to the induction of DNA damage and impaired DNA repair through the suppression of RAD51, a key homologous recombination (HR) protein [93]. The ectopic expression of RAD51 partially compromised the cytotoxic effect elicited by the cotreatment with JQ1 and HDACi, indicating that RAD51 downregulation could be significant for clinical benefit. The strong synergy between HDACis and BETis in several cancer cells motivated the design and synthesis of dual BET/HDAC inhibitors (reviewed in [94]). Similar to dual BET/HDAC inhibitors, other single-molecule cotargeting BET and cancer key drivers have been reported, including dual PI3K/BET, CDK/BET, JAK2/BET, PLK1/BET and EGFR/BET inhibitors (reviewed in [95]). Recently, the multitarget inhibition of CDK4/6-PI3K-BET with a rationally designed compound SRX3177 demonstrated broad cytotoxic activity against various cancer types [96].

A drug combination screen of 20 well-characterised drugs targeting seven classes of epigenetic regulators identified a strong synergy between BETis and PARP inhibitors (PARPis) [35]. PARPis have been extensively used to evoke synthetic lethality in cells with inefficient HR, primarily due to germline loss-of-function mutations in either *BRCA1* or *BRCA2* in breast, ovarian, prostate and pancreatic cancers [97,98]. PARPis target PARP1 and PARP2, which are necessary sensors of DNA damage that recognise single-stranded breaks (SSBs) and transduce the signal in the DDR pathway. Mechanistically, blocking PARP1’s enzymatic activity compromises the repair of SSBs, which become converted to double-stranded breaks (DSBs) during DNA replication, thus inducing synthetic lethality in cancer cells with deficient HR. BETis caused epigenetic loss of *BRCA1* expression in BRCA1 wild-type TNBC cells and synergised with PARPis impairing HR and thus triggering synthetic lethality [99]. The independence from intrinsic HR status is consistent with the BETi-induced deficiency in HR. Simultaneous inhibition of BET and PARP could expand the spectrum of cancer types qualifying for PARPi treatment beyond those with deficient HR [35,100,101].

Moreover, BETis reversed multiple mechanisms of acquired PARPi resistance, which frequently develops in patients [100]. The effects of PARPi and BRD4i combinations were observed in numerous cancer lineages, suggesting that the synergistic activity is likely to be generalisable. The synergistic effect of BETi and PARPi was attributed directly to the loss of BET and PARP1, as synergy was confirmed when four different BETis were applied or when PARP1 was knocked down. Furthermore, the synergy with PARPis was noted with BRD4 knockdown, but not with BRD2 or BRD3. The synergistic effect of BETis and PARPis was attributed to CtIP’s (an HR pathway protein) decreased transcription since forced expression of CtIP, but not BRCA1 or RAD52, rescued cells from the synergistic effect of these inhibitors.

**Table 2:** Combinations of BET inhibitors with anticancer drugs in preclinical tumour models and clinical trials.

Classes of Compounds	Compounds	Preclinical Study		Clinical Trial		
		Cancer	References	Phase/Status	Cancer	References
Small molecular weight inhibitors	ALK inhibitors	Lymphoma	[57]			
	BTK inhibitors	Lymphoma	[57,89,100,102,103,104,105,106,107]			
	CDK inhibitors	Lymphoma	[105,106]			
		Osteosarcoma	[108]			
	BCL2/MCL1 inhibitors	ALL	[109]	I/not yet recruiting	MF	NCT04480086
		AML	[62,64,110]	I, completed	Lymphoma	NCT03255096
		LC	[111]	I, completed	Advanced solid tumours and haematological malignancies	NCT02391480
		Lymphoma	[105,106,112,113,114]	I, recruiting	MF	NCT04454658
	EGFR/ERBB2 inhibitors	BC	[115]			
	FLT3/ERBB2 inhibitors	AML	[116]			
	Hedgehog inhibitors	Lymphoma	[117]			
	JAK inhibitors	AML	[118]	I/II, recruiting	MF	NCT02158858
		MF	[66,67]	I, not yet recruiting	MF	NCT04480086
				I, recruiting	MF	NCT04454658
				I/II, terminated	Solid tumours	NCT02711137
	MEK/ERK inhibitors	AML	[119]	I/II, withdrawn	Solid tumours	NCT03266159
		BC	[119]			
		CRC	[119,120]			
		Lymphoma	[89]			
		MM	[119]			
		Neuroblastoma	[121]			
		NSCLC	[110]			
		OC	[81]			
		PrC	[119]			
		Thyroid cancer	[122]			
	mTOR inhibitors	BC	[123]			
		Glioblastoma	[124]			
		Lymphoma	[89,102,103,104,107]			
		OS	[125]			
	PARP inhibitors	BC	[99,100,126]	I/II, terminated	Solid tumours	NCT02711137
		Bladder cancer	[100]			
		Endometrial cancer	[100]			
		LC	[127]			
		OC	[35,100,101]			
		PC	[100]			
	PI3K inhibitors	BC	[81,126]			
		CRC	[126]			
		Lymphoma	[102,104,128]			
		Glioblastoma	[126]			
		OC	[80,126]			
	PIKK inhibitors	Lymphoma	[129]			
	Proteasome inhibitors	MM	[62,130]			
Antibodies	Anti-CD20 monoclonal antibodies	Lymphoma	[104,107,131]			
Immune modulators	Immunomodulatory drugs (IMiDs)	Lymphoma	[89,104,132,133]			
		MM	[134]			
	Anti-PD-1 monoclonal antibodies	Lymphoma	[135]	I/II, not yet recruiting	Solid tumours and haematological malignancies	NCT02419417
				II, recruiting	Metastatic CRPC	NCT04471974
				I, not yet recruiting	Solid tumours	NCT04840589
				I/II, active, not recruiting,	Advanced tumours	NCT02419417
	Anti-4-1BB monoclonal antibodies	Lymphoma	[135]			
	Chimeric antigen receptor (CAR) T-cells	ALL	[136]			
Epigenetic drugs	EZH2 inhibitors	Lymphoma	[103,137]			
	HDAC inhibitors	AML	[116]	I/II withdrawn	Advanced and refractory solid tumours and lymphomas	NCT03925428
		Bladder cancer	[138,139]			
		BC	[140]			
		Chondrosarcoma	[93]			
		Glioblastoma	[141]			
		LC	[142]			
		Lymphoma	[57,104,106,107,113,143,144,145]			
		Melanoma	[146]			
		Neuroblastoma	[147]			
		PC	[148,149,150]			
		Sarcoma	[151]			
	Azacytidine	AML	[62]	I/II, withdrawn	AML	NCT02303782
				I/II completed	Haematological malignancies	NCT02543879
				I/II, terminated	Solid tumours	NCT02711137
	Decitabine	Lymphoma	[104]			
Chemotherapy	Gemcitabine			I/II, terminated	Solid tumours	NCT02711137
	Paclitaxel			I/II, terminated	Solid tumours	NCT02711137
	Temozolomide	Glioblastoma [124]				
Hormone therapy	Antiandrogen	PrC [152]		I/II, active, completed	PrC	NCT02711956
				II, recruiting	PrC	NCT04471974
				I/II, terminated	PrC	NCT02607228
				I/II, terminated	Solid tumours	NCT02711137
	Estrogen receptor degrader	BC [153]		I, completed	BC	NCT02392611
				I/II, terminated	BC	NCT02983604

Abbreviations: ALL, acute lymphoblastic leukaemia; AML, acute myeloid leukaemia; BC, breast cancer; CRC, colorectal cancer; CRPC, castration-resistant prostate cancer LC, lung cancer; MF, myelofibrosis; MM, multiple myeloma; OC, ovarian cancer; PC, pancreatic cancer, PrC; prostate cancer.

## 8. BET in DNA Repair

As mentioned earlier, the anticancer activity of BETis could not be merely explained by their influence on the transcription of cancer driver genes, such as *MYC.* The promising preclinical studies on the combinatorial treatment of BETis and PARPis triggering synthetic lethality in cancer cell lines of various origin imply that suppression of DNA repair efficacy could be an essential mechanism of BETis’ anticancer effect.

Alterations in DDR can lead to the accumulation of DNA damage, which is one of the main drivers of cancer progression. Moreover, insufficient DNA repair can contribute to cancer aggressiveness and can facilitate the emergence of resistance to anticancer DNA-damaging drugs [154]. Although DNA damage is a causal factor for carcinogenesis, it can be utilised in favour of anticancer treatment. Evidence indicates that inhibitors of DNA repair pathways can work as single agents for the targeted treatment of DNA repair-defective cancers [97,98]. The inhibitors of DNA damage repair target fast-replicating cells; thus, they could prove selective for cancer cells and have fewer side effects. DNA damage misrepaired or left unrepaired could persist into the S-phase of the cell cycle and result in a stalled replication fork, leading to the formation of replication-associated DSBs, generally considered to be one of the most deleterious DNA lesions. DSBs are repaired by a few DNA repair pathways, mainly HR—which requires the presence of complementary strand and thus acting in the S and G2 cell cycle phases—and non-homologous end joining (NHEJ)—which involves microhomology and is active in each cell cycle phase. The NHEJ pathway can introduce small insertions or deletions that frequently manifest as missense or frameshift mutations after repair. Apart from the principal HR and HNEJ pathways, DSBs can also be repaired by highly mutagenic alternative end-joining (alt-EJ) (also known as microhomology-mediated end joining, MMEJ), single-stranded annealing (SSA), synthesis-dependent strand annealing (SDSA) and break-induced repair (BIR). Despite the specificity of each pathway for particular DNA damage and cell cycle phase, none of them is mutually exclusive, and they form a network that involves proteins determining the repair outcomes.

**Figure 3:**
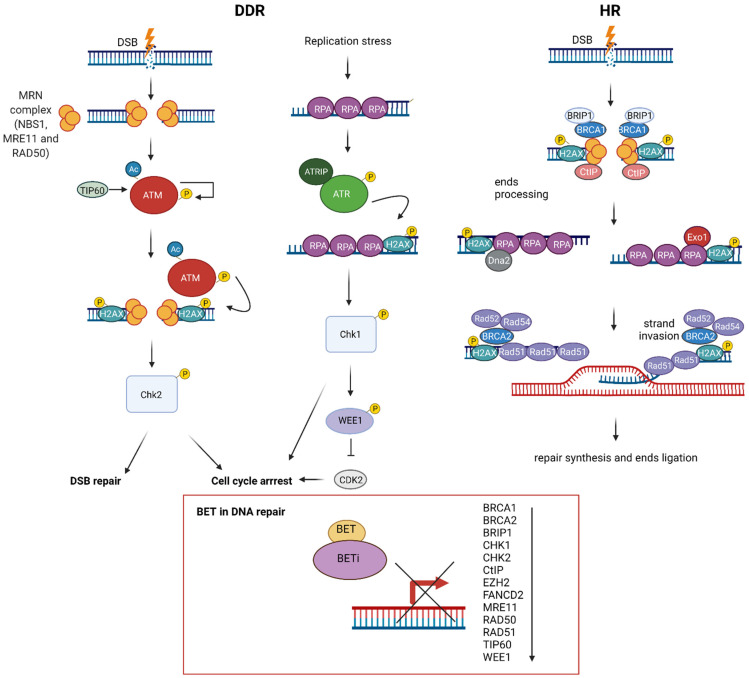
The role of bromodomain and extraterminal domain (BET) proteins in DNA damage response (DDR). DNA double-stranded breaks (DSB) are recognized by the MRN complex (NBS1, MRE11 and RAD51) and ATM/ATR kinases, followed by H2AX phosphorylation and activation of CHK1/CHK2 kinases, leading to diverse cellular responses, including cell cycle arrest and DNA repair. DSBs are mainly repaired through homologous recombination (HR), a multistep process encompassing DNA damage recognition, DNA end resection, strand invasion, repair synthesis and end ligation. BET controls key HR proteins’ transcription: BRCA1, RAD51 and CtIP, through interaction with their promoter regions. BRCA1,BRCA2, BRIP1, CtIP, FANCD2, CHK1, CHK2, MRE11, RAD50, RAD51, TIP60 and WEE1 expression is sensitive to BET inhibitors (BETis).

Mechanistic studies have revealed that BET proteins play a role in DSB repair via modulating HR, which could be beneficial for anticancer treatment (Figure 3). A first notion of the role of BET in HR repair came from the three independent studies on BETis, JQ1 and GSK525762A [35,99,100]. These studies showed that BETis inhibited BRD2/3/4 and BRDT and consequently decreased HR repair efficiency. These results were further confirmed by the pooled siRNAs targeting BRD2, BRD3 and BRD4, ensuring that BET proteins participate in HR repair [35]. Although Mio et al. and Sun et al. studied only the influence of BRD4, the research conducted by Yang et al. in 2017 demonstrated that the inhibition of each BRD2/3/4 disrupted HR.

Further analyses indicated that JQ1 impaired HR by decreasing the foci formation of BRCA1 and RAD51 in irradiated cells [35,99]. This effect was accompanied by a reduction of the mRNA and protein expression of BRCA1 and RAD51 for individual siRNAs targeting BRD2/3/4. However, it was the most prominent for pooled siRNA, indicating BET’s joint participation in transcriptional repression [35]. The importance of BET-mediated regulation of BRCA1 and RAD51 expression in carcinogenesis should be emphasised because their expression was repressed by BETis to a comparable extent as MYC repression in multiple cancer models. BRCA1 and RAD51 colocalise at nuclear foci [155] and interact with each other to provide key steps of HR. BRCA1 participates in DNA end resection, providing single-strand DNA (ssDNA), a prerequisite and determinant step for HR. After the generation of ssDNA, BRCA1 recruits other proteins, including RAD51, to DSB sites. RAD51 is a recombinase that forms filaments with ssDNA to conduct homology search and strand invasion [156]. A recent study demonstrated that BRCA1 stimulates the recombinase activity of RAD51 and promotes RAD51-mediated pairing of homologous sequences [157]. Considering the critical role of BET proteins in gene transcription, the effect of BETis on the transcription of BRCA1 and RAD51 was investigated. A mechanistic study revealed that JQ1 repressed the expression of BRCA1 and RAD51 by reducing the recruitment of BRD2/3/4 to their promoter regions [35,99]. Furthermore, a super-enhancer region in the *BRCA1* gene was identified [35]. It was shown that this BET-sensitive super-enhancer region physically interacts with the *BRCA1* promoter, influencing BRCA1 expression.

On the other hand, Sun et al. demonstrated that four different BETis decreased the expression of essential DNA repair genes, among which CtIP was the most suppressed [100]. Among all deregulated DNA repair proteins, CtIP was strongly and consistently downregulated under all conditions, while the effects of BETis on RAD51 and BRCA1 were modest and variable. Moreover, JQ1 repressed BRD4 binding to the *CtIP* promoter and enhancer and decreased the association of Pol II with these sequences. CtIP is an endonuclease that cooperates with the MRE11-RAD50-NBN (MRN) complex in DNA end resection. Its BETi-mediated downregulation was sufficient to impair HR function. The overexpression of CtIP, but not RAD51 or BRCA1, partially rescued BRD4-inhibition-induced defects in DNA end resection and HR.

Apart from repressing BRCA1, RAD51 and CtIP expression, BETis downregulated the expression of HR proteins such as BRCA2, BRIP1, FANCD2, CHK1, CHK2, MRE11, RAD50 TIP60, WEE1 and EZH2 [89,93,99,100]. BRCA2 is the primary mediator of RAD51 nucleofilament formation and strand exchange in HR. BRIP1 (FANCJ/BACH1) is a DEAH helicase that interacts with the BRCT domain of BRCA1 and plays a role in HR and the repair of crosslinks by the Fanconi anaemia pathway [158,159]. FANCD2 colocalises with BRCA1, BRCA2 and RAD51 nuclear foci and is involved in DSB and crosslinks repair [160,161,162]. CHK1/2 are kinases that orchestrate DDR [163]. MRE11 and RAD50 are components of the MRN complex, which together with CtIP participate in DNA resection in HR [164]. TIP60 is a HAT that regulates HR repair [165]. WEE1 is a checkpoint kinase that inhibits Cdc2, resulting in G2 arrest [166]. EZH2 is a histone-lysine *N*-methyltransferase that regulates the expression of *BRCA1* and *RAD51* [167]. Overall, these results indicate that BETis decrease HR. On the other hand, JQ1 treatment did not influence the expression of the Ku complex, which plays a crucial role in NHEJ. Additionally, JQ1 increased NHEJ efficiency, indicating that BETis switched the repair of DSB from HR to NHEJ [35]. Indeed, the comparative analysis demonstrated HR deficiency in cells with individual siRNA-mediated knockdown of BRD2/3/4, while NHEJ stayed intact [168]. On the other hand, BRD4 was critical for NHEJ in the class switch recombination (CSR) occurring in B-cells [169]. Moreover, JQ1 was sufficient to suppress CSR, indicating that NHEJ repair and CSR are directly dependent on the presence of BRD4. 

**Figure 4:**
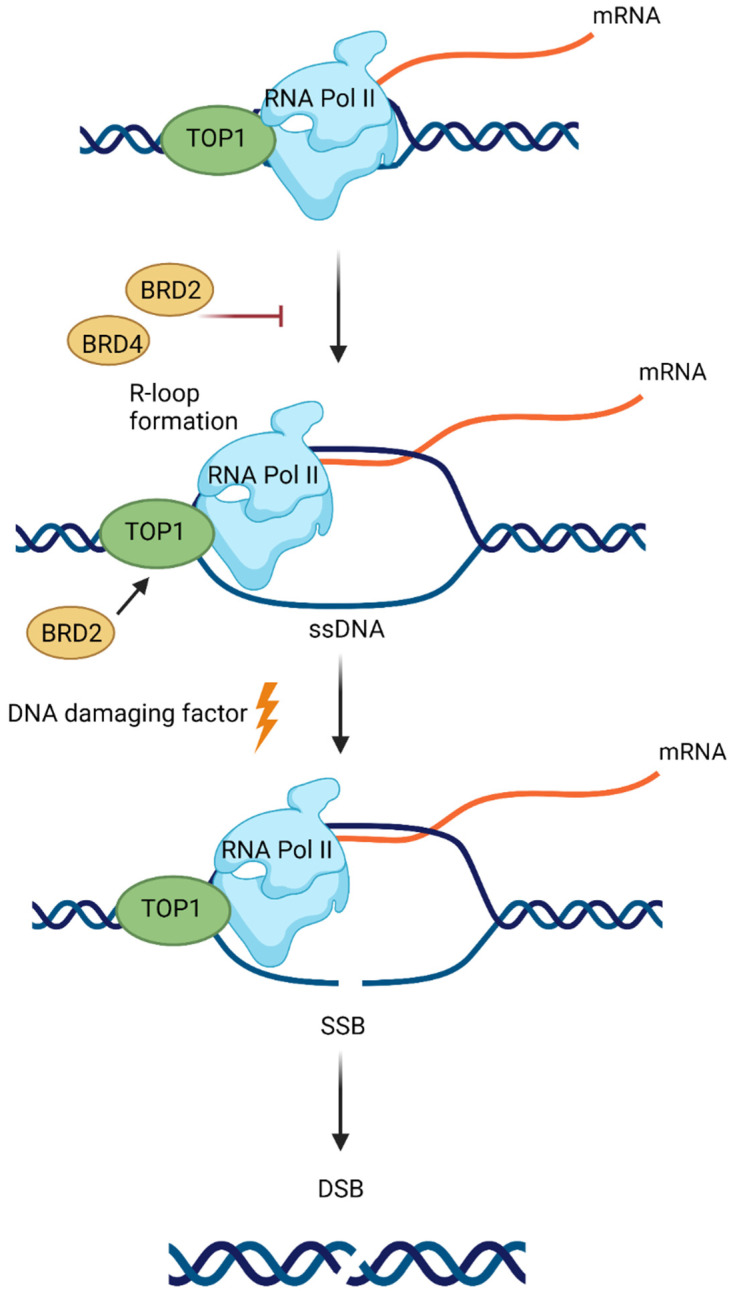
Bromodomain and extraterminal domain 2 and 4 (BRD2 and BRD4) proteins prevent DNA damage induction at the sites of RNA polymerase II (RNA Pol II) activity. BRD2 and BRD4 restrict the formation of transcription-associated DNA:RNA hybrids (R-loops). Topoisomerase I (TOP1) activity, a known restrainer of R-loops, is promoted by BRD2. R-loop formation leads to the exposure of a single-strand DNA (ssDNA) on the nontemplate strand to DNA damaging factors. This can result in the induction of single-stranded breaks (SSBs), which can be converted to double-stranded breaks (DSBs).

**Figure 5:**
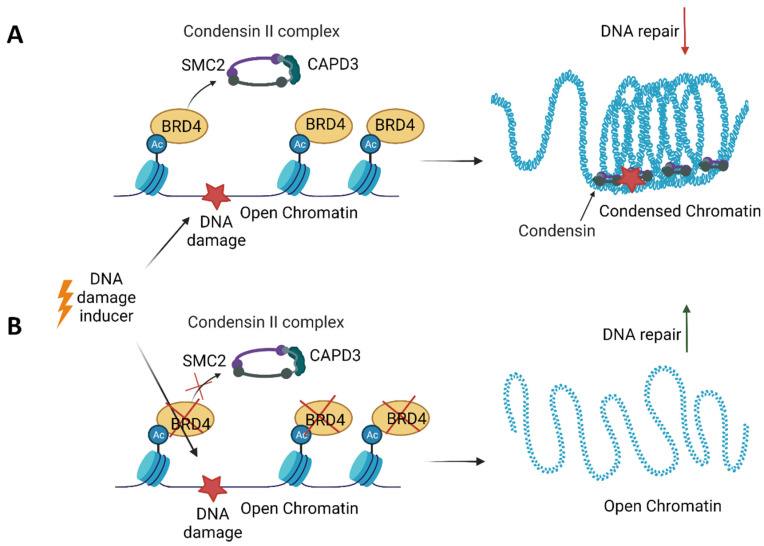
An isoform B of bromodomain and the extraterminal domain 4 (BRD4) protein participates in the persistence of DNA damage. (**A**) BRD4 binds acetylated histones in the open chromatin regions. Upon DNA damage, BRD4 recruits the condensin II complex consisting of SMC2 and CAPD3 to acetylated chromatin, resulting in chromatin compaction, attenuation of DNA repair and the persistence of DNA damage. (**B**) In the absence of BRD4, the chromatin remains open, allowing for DNA repair upon DNA damage.

Additionally, BETis directly influenced DDR by increasing the formation of γH2AX, a marker of DSBs (Figure 4) [25,93,100,168]. Specific depletion of BRDT, BRD2, BRD3 and BRD4 indicates that BRD2 and BRD4 knockdown increased DSBs levels, suggesting that cells deficient in these BETs are susceptible to spontaneous DSB formation [168]. Given that BET-deficient cells are characterised by altered transcription and elevated DSBs, the role of transcription in DNA damage induction was investigated. BRD2 and BRD4 inhibited the formation of transcription-associated RNA-DNA hybrids (R-loops). A mechanistic study revealed that BRD2 promoted topoisomerase I activity, a known restrainer of R-loops, thus explaining the increased R-loop formation and subsequent endogenous DSBs’ induction observed in BRD2-deficient cells. Another mechanism that contributes to increased DNA damage formation in BETi-treated cells involves the role of BRD4. An isoform B of BRD4 was identified to recruit the condensin II complex, SMC2 and CAPD3, to the sites of acetylated regions of chromatin in response to DNA damage (Figure 5) [25]. BRD4-mediated chromatin condensation resulted in DDR attenuation. BETis reversed this process leading to a more open chromatin structure that facilitates γH2AX foci formation. 

Besides γH2AX, BET proteins interacts with other critical DNA-damage-signalling proteins. The DDR signalling pathway is orchestrated by the ATM and ATR kinases, which recognise DNA damage and activate downstream kinases. The second wave of phosphorylation is conducted by Chk1 and Chk2, predominantly via the ATM-Chk2 and ATR-Chk1 cascades. BETis synergise with ATR and Chk1 inhibitors in *MYC*-dependent lymphoma cells and are associated with increased DSBs and the induction of apoptosis [89,129].

## 9. Conclusions and Future Perspectives

Recent recognition of the role of transcriptional deregulation in cancer initiation and progression has led to the appreciation of transcriptional apparatus inhibitors [170]. Dysregulation of gene expression programs in cancer cells can occur through dysregulation of oncogenic master transcription factors, dysregulation of signalling and dysregulation of a transcriptional amplifier, such as MYC. Given that most human cancers exhibit genetic amplification or transcriptional dysregulation of MYC, the pharmacologic inhibition of MYC has been highly anticipated. MYC’s direct pharmacologic inhibition is challenging because transcription factor oncoproteins have been mainly refractory to conventional drug discovery approaches. Hitherto “undruggable” MYC has been indirectly targeted through the inhibition of BET, which are epigenome-regulating factors. 

Eleven years have passed since the synthesis and biological evaluation of the first BETi, JQ1. Several BETis underwent ~35 ongoing or completed clinical trials during this period, while many others are currently in the drug development program. BETis have been tested mostly against MYC-dependent cancers, including hematologic malignancies such as B-cell lymphoma, AML, MM and solid tumours, such as brain, colorectal, lung, prostate and breast cancers. The anticancer properties seem to reflect the inhibition of BETi-mediated regulation of *MYC* expression. Contrary to the above mechanism of action, MYC-independent sensitivity to BETis was reported, suggesting that BETs target other proteins crucial for carcinogenesis [108,171]. 

Unfortunately, despite extensive preclinical and clinical evaluation, none of the BETis has yet received regulatory approval. Apart from NMC, no predictive biomarker has been identified so far, indicating that these trials are not targeting specific molecular subtypes (reviewed in [54]). Predictive biomarkers are essential for (1) selecting patients with the characteristics of inhibitor sensitivity, (2) verifying target inhibition and (3) understanding resistance mechanisms [172]. The lack of a biological rationale for BETi-oriented anticancer therapy hinders the introduction of BETis into regular clinical practice. 

The advances in the development of specific BETis have drawn attention in medicinal chemistry as much as the identification of predictive biomarkers is crucial in molecular oncology. The emphasis is now on the design of selective inhibitors targeting either BET or BD1—a domain whose inhibition has demonstrated anticancer potential.

Based on the conducted clinical trials, it seems that BETis might rather be exploited in targeted therapies in selective cancer types than in conventional chemotherapy [173]. Clinical activity has been observed in NMC, haematological malignancies, including MM, AML, lymphomas and MLL, and solid tumours such as TNBC and CRPR, nonsmall-cell lung carcinoma or glioblastoma. Concerns about applying BETis in patients focus on their ability to specifically target cancer cells without affecting the homeostasis of normal cells. BETis primarily affect the epigenome landscape and thus the expression of transcription factors, including MYC. Although inhibition of transcription factors is presumed to act widely on numerous genes, it can exert highly selective effects on gene expression control [170]. Targeting tissue-specific master transcription factors in cancer, such as the oestrogen receptor, progesterone receptor and androgen receptor, has profound clinical benefit in treating hormone-dependent breast and prostate cancer. However, the knowledge of the clinical outcome of a few transcription factors cannot be directly translated to all transcription factors as the knowledge of tissue-specific master transcription factors remains limited. Other than BETis, epigenome-targeting drugs, such as HDACis, have been approved by the U.S. Food and Drug Administration (FDA) for oncological indications, raising hope that epigenetic drugs could be successfully used in anticancer treatment. 

Multiple resistance mechanisms and DLTs further limit BETis’ efficacy, demonstrating the need for the use of BETis in combination therapy. The improved anticancer efficacy of BETis was noted in combination with different classes of compounds. Among them, the combination with inhibitors of DNA repair has been promising in preclinical evaluation. BETis induce HR deficiency through multiple mechanisms, including downregulation of RAD51, BRCA1 and CtIP, presumably via binding to their promoter regions. Additionally, these HR pathway proteins are dependent on MYC, which binds to the promoters of several HR pathway genes and transcriptionally regulates multiple components of the HR repair pathway, suggesting that BETis might have therapeutic potential suppressing HR directly and indirectly [174,175,176,177,178]. The dual inhibition of BETis and PARPis demonstrated synthetic lethality. Hopefully, as a few PARPis have received FDA approval so far, with a high probability, more approvals are coming.

In conclusion, BETis have potential as anticancer drugs; however, their clinical progress faces significant obstacles. It is expected that the advances in the development of next-generation compounds, the identification of predictive biomarkers and combination therapy will allow for BETis regulatory approval. Undoubtedly, research on BETis will contribute to a greater understanding of cancer biology, genomics and epigenomics.

## Figures and Tables

**Table 1:** BET inhibitors in clinical trials.

Compound	Target	Combination	Tumour Type	Results	Phase/Status	Reference
ABBV-075	BRD2/4, BRDT	N/A	Solid tumours: BC, CRC, PR, PrC, uveal melanoma, head and neck	SD: 26 *n* = 71	I, completed	NCT02391480 [53]
		Venetoclax	AML	CR: 3 PR: 2 MLFS: 2 *n* = 44		[64]
		Navitocla xRuxolitinib	MF	Not posted	I, not yet recruiting	NCT04480086
ABBV-744	BRD2/3/4, BRDT	N/A	AML	Not posted	I, terminated	NCT03360006
		Navitocla xRuxolitinib	MF	Not posted	I, recruiting	NCT04454658
BAY1238097	BRD4	N/A	Solid tumours, myeloma, lymphoma	NR: 8 SD: 2 *n* = 11	I, terminated	NCT02369029 [61]
BI 894999	BRD2/3/4, BRDT	N/A	Solid tumours and lymphoma	Not posted	I, not yet recruiting	NCT02516553
BMS-986158	Undisclosed	N/A	Solid tumours and lymphoma in children	Not posted	I, recruiting	NCT03936465
		Nivolumab	Advanced solid tumours and haematological malignancies	Not posted	I/II, not yet recruiting	NCT02419417
CC-90010	Undisclosed	N/A	Solid tumours and NHL	Not posted	I, recruiting	NCT03220347
CPI-0610	BRD4	N/A	Lymphoma	CR: 2 PR: 3 SD: 5 *n* = 64	I, completed	NCT01949883 [65]
		N/A	MM	Not posted	I, completed	NCT02157636
		N/A	Peripheral nerve tumours	Not posted	II, withdrawn	NCT02986919
		Ruxolitinib	MF	Not posted	I/II, recruiting	NCT02158858 [66,67]
FT-1101	BRD2/3/4, BRDT	Azacitidine	AML, MDS, NHL	Not posted	I, completed	NCT02543879
GS-5829		N/A	Solid tumours, lymphoma	Not posted	I, completed	NCT02392611
		Exemestane Fulvestrant	ER-positive BC	Not posted		
		Exemestane Fulvestrant	ER-positive and HER-negative BC	Not posted	I/II, terminated	NCT02983604
		Enzalutamide	Castrate-resistant PrC	Not posted	I/II, terminated	NCT02607228
I-BET151 (GSK2820151)	BRD2/3/4	N/A	Solid tumours	Not posted	I, terminated	NCT02630251
I-BET762 (GSK525762)	BRD2/3/4, BRDT	N/A	Hematologic malignancies	Not posted	I/II, completed	NCT01943851
		N/A	NMC	PR: 2 SD: 7 *n* = 19	I, completed	NCT01587703 [53,68]
		Trametinib	Solid tumours	Not posted	I/II, withdrawn	NCT03266159
		Entinostat	Solid tumours and haematological malignancies	Not posted	I, withdrawn	NCT03925428
INCB054329	BRD2/3/4, BRDT	N/A	Solid tumours and haematological malignancies	Not posted	I/II, withdrawn	NCT02431260
INCB057643	BRD2/3/4	Abiraterone, Azacitidine, Gemcitabine, Paclitaxel, Rucaparib, Ruxolitinib	Solid tumours	CR: 2 PR: 4 *n* = 134	I/II, terminated	NCT02711137 [69]
ODM-207	Undisclosed	N/A	Solid tumours	SD: 6 *n* = 27s	I/II, completed	NCT03035591 [70]
OTX015 (MK-8628)	BRD2/3/4	N/A	AML, DLBCL	Not posted	I, active, not recruiting	NCT02698189
		N/A	CRPC, NMC, NSCLC, TNBC	Not posted	I, terminated	NCT02698176
		N/A	Glioblastoma multiforme	Not posted	II, terminated	NCT02296476
		N/A	CRPC, NMC, NSCLC, PC, TNBC	PR: 3 (NMC) SD: 25 (3 NMC) *n* = 46	I, completed	NCT02259114 [52] [50]
		N/A	AML, acute lymphoblastic leukaemia, DLBCL, MM	CR:2 (DLBCL) PR:1 (DLBCL) *n* = 33 CR: 2 (AL) PR: 3 (AL) *n* = 41	I, completed	NCT01713582 [47] [55]
		Azacitidine	AML	Not posted	I/II, withdrawn	NCT02303782
PLX51107	BRD2/3/4, BRDT	N/A	Solid tumours and haematological malignancies	Not posted	I/II, terminated	NCT02683395
RO6870810 (TEN-010)	Undisclosed	N/A	AML, MDS	CR: 1 SD: 13 *n* = 32	I, completed	NCT02308761 [71]
		N/A	Solid tumours	PR: 1 (solid tumours) SD: 24 (solid tumours) *n* = 47 PR: 2 (NMC) SD: 5 (NMC) *n* = 8 PR: 2 (DLBCL) SD: 4 (DLBCL) *n* = 19	I, completed	NCT01987362 [72]
		Venetocla xRituximab	DLBCL, B-cell lymphoma	Not posted	I, completed	NCT03255096
ZEN-3694	Undisclosed	N/A	Metastatic CRPC	Not posted	I, completed	NCT02705469
		Enzalutamide	Metastatic CRPC	Not posted	I/II, completed	NCT02711956
		Enzalutamide Pembrolizumab	Metastatic CRPC	Not posted	II, recruiting	NCT04471974
		Ipilimumab Nivolumab	Solid tumours	Not posted	I, not yet recruiting	NCT04840589

Abbreviations: AL, acute leukaemia; AML, acute myeloid leukaemia; CR, complete response; CRC, colorectal cancer; CRPC, castrate-resistant prostate cancer; DLBCL, diffuse large B-cell lymphoma; MDS, myelodysplastic syndrome; MF, myelofibrosis; MLFS, morphological leukaemia-free state; MM, multiple myeloma; NHL, non-Hodgkin’s lymphoma; NMC, NUT midline carcinoma; NR, no response; PC, pancreatic cancer; PR, partial response; SD, stable disease; TNBC, triple-negative breast cancer. Additional information: abiraterone (AR pathway inhibitor), azacitidine (DNMT inhibitor), entinostat (HDAC inhibitor), enzalutamide (AR inhibitor), exemestane (ER pathway inhibitor), fulvestrant (ER inhibitor), gemcitabine (cytostatic drug), ipilimumab (anti-CTLA-4 antibody), navitoclax (BCL2 inhibitor), nivolumab (PD-1 inhibitor), paclitaxel (cytostatic drug), pembrolizumab (anti-PD-1 antibody), rituximab (anti-CD20 antibody), rucaparib (PARP inhibitor), ruxolitinib (JAK inhibitor), trametinib (MEK inhibitor), venetoclax (BCL2 inhibitor).

## Data Availability

All important data is included in the manuscript.
